# Pancreatic Cancer Chemotherapy Is Potentiated by Induction of Tertiary Lymphoid Structures in Mice

**DOI:** 10.1016/j.jcmgh.2021.06.023

**Published:** 2021-07-09

**Authors:** Francesca R. Delvecchio, Rachel E.A. Fincham, Sarah Spear, Andrew Clear, Marina Roy-Luzarraga, Frances R. Balkwill, John G. Gribben, Michele Bombardieri, Kairbaan Hodivala-Dilke, Melania Capasso, Hemant M. Kocher

**Affiliations:** 1 Centre for Tumor Biology, Barts Cancer Institute, Cancer Research UK Barts Centre, Queen Mary University of London, London, United Kingdom; 2 Centre for Experimental Medicine and Rheumatology, William Harvey Research Institute, Queen Mary University of London, London, United Kingdom; 3 Centre for Tumor Micro-environment, Barts Cancer Institute, Cancer Research UK Barts Centre, Queen Mary University of London, London, United Kingdom; 4 Centre for Haemato-Oncology, Barts Cancer Institute, Cancer Research UK Barts Centre, Queen Mary University of London, London, United Kingdom; 5 German Centre for Neurodegenerative Diseases, Bonn, Germany

**Keywords:** B Cells, T Cells, Dendritic Cells, Orthotopic, Transgenic Mice, BCL6, B cell lymphoma 6, BMDC, bone marrow–derived dendritic cell, FDC, follicular dendritic cell, HEV, high endothelial venules, PBS, phosphate-buffered saline, PBS-T, Tween-20 in phosphate-buffered saline, PDAC, pancreatic ductal adenocarcinoma, RT, room temperature, SLO, secondary lymphoid organ, TLS, tertiary lymphoid structures, TMA, tissue microarray

## Abstract

**Background and aims:**

The presence of tertiary lymphoid structures (TLSs) may confer survival benefit to patients with pancreatic ductal adenocarcinoma (PDAC), in an otherwise immunologically inert malignancy. Yet, the precise role in PDAC has not been elucidated. Here, we aim to investigate the structure and role of TLSs in human and murine pancreatic cancer.

**Methods:**

Multicolor immunofluorescence and immunohistochemistry were used to fully characterize TLSs in human and murine (transgenic [KPC (*Kras*^*G12D*^, *p53*^*R172H*^, *Pdx-1-Cre*)] and orthotopic) pancreatic cancer. An orthotopic murine model was developed to study the development of TLSs and the effect of the combined chemotherapy and immunotherapy on tumor growth.

**Results:**

Mature, functional TLSs are not ubiquitous in human PDAC and KPC murine cancers and are absent in the orthotopic murine model. TLS formation can be induced in the orthotopic model of PDAC after intratumoral injection of lymphoid chemokines (CXCL13/CCL21). Coadministration of systemic chemotherapy (gemcitabine) and intratumoral lymphoid chemokines into orthotopic tumors altered immune cell infiltration ,facilitating TLS induction and potentiating antitumor activity of chemotherapy. This resulted in significant tumor reduction, an effect not achieved by either treatment alone. Antitumor activity seen after TLS induction is associated with B cell-mediated dendritic cell activation.

**Conclusions:**

This study provides supportive evidence that TLS induction may potentiate the antitumor activity of chemotherapy in a murine model of PDAC. A detailed understanding of TLS kinetics and their induction, owing to multiple host and tumor factors, may help design personalized therapies harnessing the potential of immune-oncology.

## Graphical abstract

**Figure:**
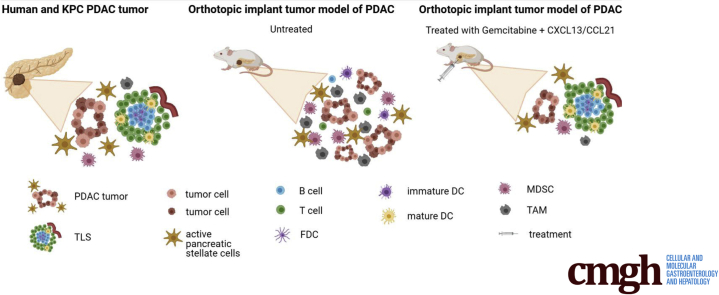

**Summary**

Intratumoral injection of lymphoid chemokines in the orthotopic tumor implantation model of pancreatic cancer could induce formation of tertiary lymphoid structures conferring therapeutic benefit, owing to tertiary lymphoid structure–associated B cells facilitating maturation of dendritic cells.

The lack of effective chemotherapy, radiotherapy, or targeted therapy combinations for advanced pancreatic ductal adenocarcinoma (PDAC) has stimulated research into immunooncology strategies,^1^ with some early success with anti-CCR2 (C-C chemokine receptor 2)^2^ and anti-PD-1 (programmed cell death protein-1) strategies in patients with a mismatch repair deficiency.^3^ Meanwhile, omics analyses have revealed multiple immune profiles among PDAC patients. Immunogenic subsets of patients with PDAC exhibit enrichment in genes associated with B cell signaling, CD4^+^ and CD8^+^ T cell infiltration, and antigen presentation. These patients may have a survival advantage over other subtypes,^4^^,^^5^ such as “immune-escape” subtypes, which exhibit a paucity of T and B cells and enrichment in FoxP3^+^ T regulatory cells.^6^^,^^7^ Intriguingly, recent evidence suggests that a proportion of patients with PDAC may require exogenous immunogenic stimuli to trigger antitumor activity.^8^ A further, smaller proportion of patients may develop specific immune evasion despite a highly cytotoxic immune phenotype termed “immune-exhausted.”^6^^,^^7^ However, the majority of the “immune-rich” PDAC patients have an inherent immunogenic potential, with the best outcome seen among those defined by presence of immune clusters named tertiary lymphoid structures (TLSs).^6^^,^^7^ The immunogenic potential suggested by genomic data is corroborated by ex vivo histopathological analyses of human samples, which correlate the organized spatial distribution of immune cells in TLSs with better prognosis in human PDAC.^7^^,^^9^^,^^10^ TLSs, in their activated state, have been shown to support in situ immune responses.^11^^,^^12^ However, based on mere histological assessment in human PDAC, lymphoid aggregates have been described as TLSs, with near ubiquitous presence, without detailed structural or functional characterization.^9^^,^^10^ In this report, we investigated the structure and function of TLSs in human PDAC and experimental models of pancreatic cancer to evaluate their role in antitumor activity.

## Results

### Fully Formed TLSs Are Present in Human and KPC PDAC But Infrequently in Orthotopic Tumor Implantation Model

**Figure 1:**
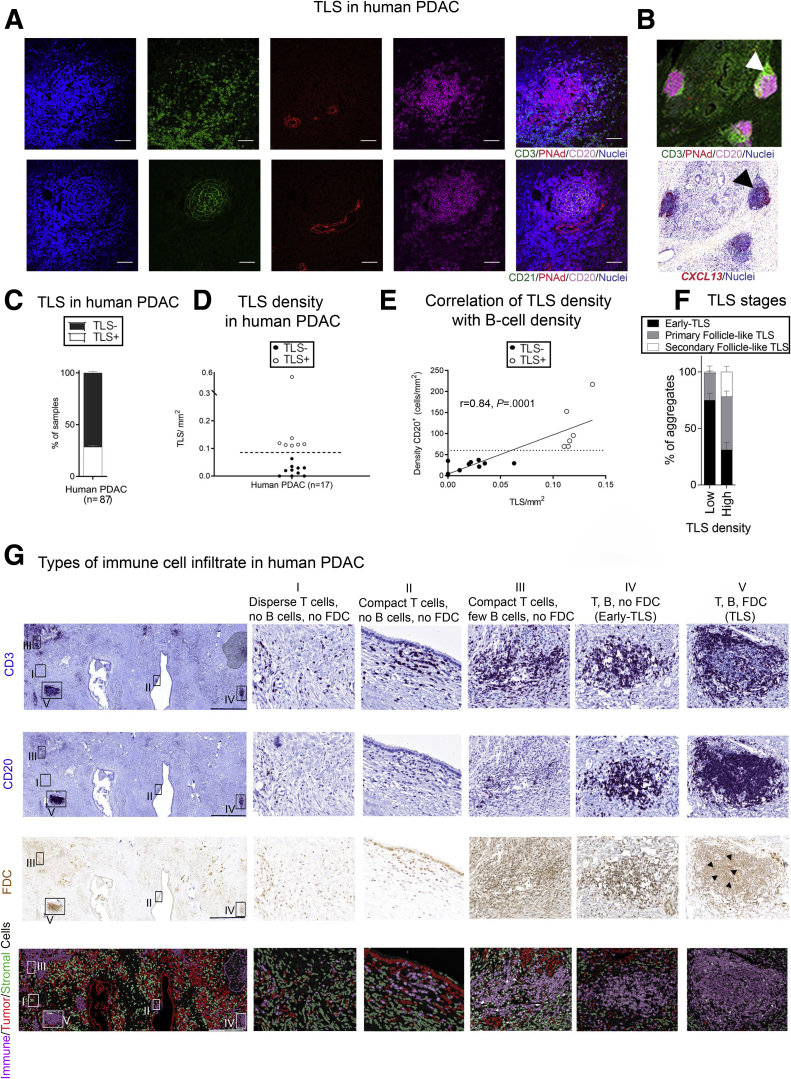
**Immunologically active TLSs are present in a fraction of chemo-naïve human PDAC.** (*A*) Colocalization of T cells (CD3^+^), B cells (CD20^+^), and FDCs (CD21^+^) with high endothelial venules (peripheral node addressin positive [PNAd^+^]) in dense, compact lymphoid aggregates on consecutive sections defining human TLSs. (*B*) Human PDAC section stained for CD20 (magenta), CD21 (green), PNAd (red), and DAPI (blue) (upper panel). Sequential section with in situ hybridization of *CXCL13* (red) RNA-scope probe in human PDAC patient (lower panel). (*C*) Frequency of TLSs in human PDAC (n = 56 TMAs, n = 31 full sections). (*D*) TLS density (expressed as number of CD3^+^CD20^+^CD21^+^ clusters/mm^2^) in a cohort of human PDAC (n = 17). The dotted line represents cutoff for identification of TLS^+^ (empty circles) and TLS^–^ (bold circles) PDAC patients. (*E*) Plot showing the significant correlation of CD20^+^ B cell density with TLS density. The dotted line represents the cutoff of minimal B cell density needed to induce ectopic lymphoneogenesis. Empty circles represent TLS^+^ patients, bold circles represent TLS^–^ patients. Spearman r = 0.84, *P =* .0001. (*F*) Distribution of TLS stages in patients with different TLS densities (TLS maturation). (*G*) Human PDAC full section stained for CD3^+^ T cells, CD20^+^ B cells, and FDCs, using modified immunohistochemistry stripping and reprobing protocol. Fourth (bottom) panel represents pseudo-color immunofluorescence image of the same sections, showing a cell phenotype map (immune, stromal, and tumor cells) using different colors to better depict spatial distribution. The boxes identify subsequent adjacent panels (I–V). Scale bar: 1000 μm. (I) Higher magnification of a representative area of presence of scattered T cells, with absence of B cells and FDCs. (II) Higher magnification of a representative area of cluster of T cells, with absence of B cells and FDCs. (III) Higher magnification of a representative area of sparse conglomerates of T cells and sparse B cells. FDCs are absent. (IV) Higher magnification of a representative area where T and B cells are clustered but not organized in distinct zones. FDCs are absent (early TLSs).(V) Higher magnification of TLSs, where a cloud of T cells surrounds a core of B cells that includes a FDC network. Those TLSs can show absence or presence of a germinal center; therefore, they are celled primary follicle-like TLSs and secondary follicle-like TLSs, respectively. Each data point represents an individual patient and lines represent median and interquartile range. Empty circles represent TLS^+^ tumors, bold circles represent TLS^–^ tumors.

TLSs were characterized based on the compact cellular organization of T cell (CD3^+^)– and B cell (CD20^+^)–rich zones, a network of follicular dendritic cells (FDCs) (CD21^+^), and presence of specific vasculature (high endothelial venules [HEV], PNAd^+^ [peripheral node addressin]) in human PDAC (Figure 1*A*).^13^ The intense expression of CXCL13 messenger RNA within the follicle-like structures indicates a possible chemotactic effect on CXCR5^+^ CD4^+^ T and B cells in patients with PDAC (Figure 1*B*). Using our stringent parameters to define TLSs as fully formed structures, rather than histological assessment or surrogate markers, we demonstrate that fully formed TLSs are not ubiquitous. They are present in 30% of tissue microarrays (TMAs) (n = 56) and 42% of full section (n = 14 and 17 in 2 cohorts) derived from chemo-naïve human PDAC samples (Figure 1*C* and *D*). Although B cells represent only a fraction of the immune cell infiltrate in PDAC, TLSs are found in patients with high B cell infiltrate (Figure 1*E*), in keeping with their recently discovered immunostimulatory phenotype when tumor infiltrating.^12^^,^^14^^,^^15^ Even though it is expected that a high number of B cells correlate with TLSs, we show that a critical mass (minimum number of 70 B cells/mm^2^) is needed in order to induce lymphoneogenesis (TLS formation) (Figure 1*E*).

Moreover, we observed different TLS phenotypes within and between patients based on their capacity to develop an FDC network and germinal center formation (Figure 1*F* and *G*). We also noted scattered or clustered T cells with no B cells (Figure 1*G*, I-II), T cell clusters with sparse B cells and no FDCs (Figure 1*G*, III), T and B cell conglomerates without compartmentalization in B/T cell–rich zones, and association with FDCs (early TLS Figure 1*G*, IV),^16^ as well as defined B cell–like follicles (Figure 1*G*, V). The observation of a predominant T cell diffuse pattern in patients with low TLS density, and the increasing prevalence of the segregation pattern in patients with higher TLS density is suggestive of a stage-wise maturation of TLSs. This is also observed in colorectal cancer and lung squamous cell carcinoma,^16^^,^^17^ with diffuse T cells, followed by B cell engagement in a sequential pattern of segregation through early and immature TLS, which eventually give rise to fully formed TLSs that may have immune activity.^16^^,^^17^ Indeed, we observed that tumors with high TLS density presented more mature TLS stages (Figure 1*F*), underscoring the need for structural characterization to define mature TLSs, as they may have a functional impact.

**Figure 2:**
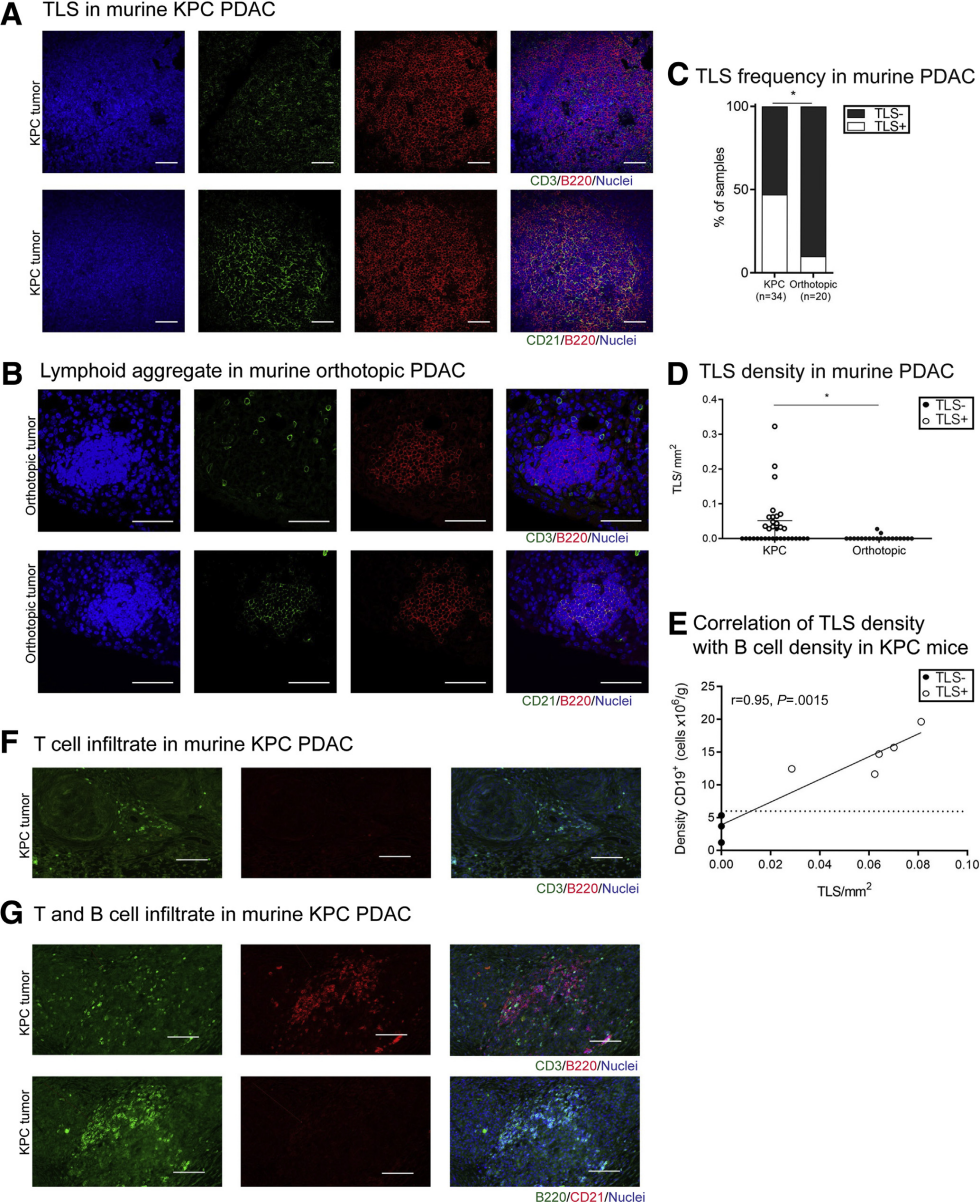
**Immunologically active TLSs are present in a fraction of KPC PDAC, similar to human PDAC, and are infrequent in orthotopic model.** (*A*) Colocalization of T cells (CD3^+^), B cells (B220^+^), and FDCs (CD21^+^) in dense, compact lymphoid aggregates in KPC tumors defining murine TLSs. (*B*) Smaller and less defined conglomerates of T (CD3^+^) and B (B220^+^) cells in orthotopic model of PDAC on consecutive sections resembling TLSs when weak presence of FDCs (CD21^+^), named lymphoid aggregates, can be detected. (*C*) TLS frequency in KPC and orthotopic murine models of PDAC. (*D*) Density of TLSs in KPC and orthotopic murine models of PDAC. (*E*) Plot showing the significant correlation of CD20^+^ B cell density with TLS density in KPC murine model of PDAC. The dotted line represents the cutoff of minimal B cell density needed to induce lymphoneogenesis. Spearman r = 0.95, *P =* .0015. (*F*) Representative immunofluorescence images of sparse T cells (CD3^+^, green) and no B cells (B220^+^, red) and FDCs, with DAPI (blue) as a nuclear stain. (*G*) Representative immunofluorescence images in sequential sections of clustered T (CD3^+^, green) and B (B220^+^, red or green) cells without compartmentalization and without FDCs (CD21^+^, red) network. Each data point represents a mouse and lines represent median and interquartile range. Empty circles represent TLS^+^ murine tumors, bold circles represent TLS^–^ murine tumors. (*C*) Chi-square test, (*D*) Mann-Whitney *U* test. ∗*P <* .05. Scale bar: 50 μm.

The paucity of functional studies on TLSs in cancer, particularly PDAC, is due to the lack of appropriate murine models.^14^ We investigated the presence of TLSs in 2 widely used murine models of PDAC, the KPC (*Kras*^*G12D*^, *p53*^*R172H*^, *Pdx-1-Cre*) genetically engineered model of PDAC, and the more cost- and time-effective orthotopic implantation of KPC-derived tumor cell line into immune-competent murine pancreas (referred to as orthotopic) (Figure 2*A* and *B*). Similar to human PDAC, in the spontaneous, autochthonous KPC mouse, the gold standard preclinical model for cognate human pancreatic cancer,^18^ TLSs were present in only 47% (n = 34) of murine tumors (Figure 2*C* and *D*) and were found to associate with high B cell infiltrate (Figure 2*E*).^14^ Akin to human PDAC, a critical mass of B cells is needed for initiation of ectopic lymphoneogenesis (Figure 2*E*). Similar to human PDAC tumors, different stages of TLS development could be identified within KPC tumors (Figure 2*F* and *G*). Conversely, orthotopic injection of a KPC-derived cell line in syngeneic wild-type mice, which produces rapid tumors within a month (and have sparse desmoplastic stroma), did not show a close-knit, organized TLS even in the 10% (n = 20) of mice where the cellular components (B cells, T cells, and sparse and weak FDC network) of TLSs were present (Figure 2*C* and *D*). We have termed these as lymphoid aggregates. Furthermore, the critical mass of B cells for initiation of the ectopic lymphoneogenesis process is never reached in the orthotopic model.^14^

### Functional TLSs in Human and KPC Tumors

**Figure 3:**
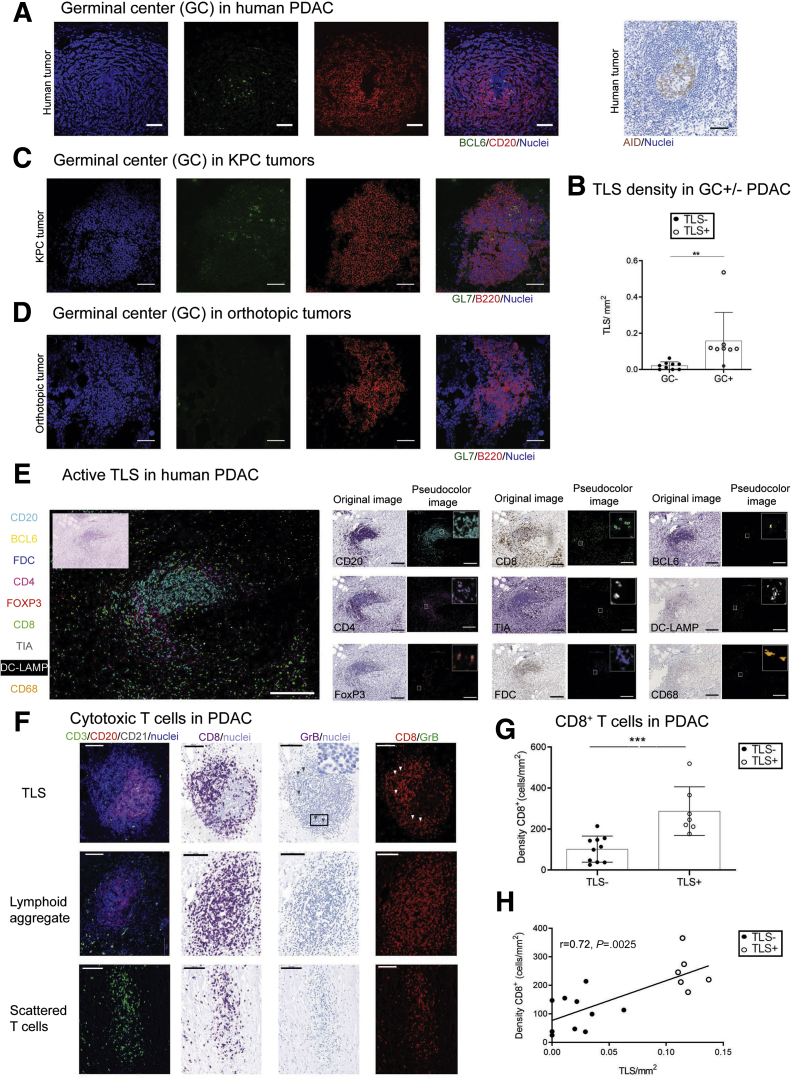
**Evidence of active TLSs in human PDAC, and presence of germinal centers in human and KPC tumors but not in orthotopic tumors.** (*A*) BCL6^+^ and AID^+^ (activation-induced deaminase) germinal centers (GCs) presence within B cell (CD20^+^) core in human PDAC. (*B*) TLS density in GC^+/–^ patients (n = 17). (*C*) GL7^+^ GCs within B cell (B220^+^) core in KPC murine model of PDAC. (*D*) Absence of GL7^+^ GCs in orthotopic PDAC mice. (*E*) Pseudo-multicolor immunophenotype of fully formed TLSs in human PDAC showing clustering of CD4^+^, CD8^+^, and TIA^+^ T cells with exclusion of Foxp3^+^ cells around a compact CD20^+^ core with BCL6^+^ B cells and a network of FDCs (FDCs, CD21^+^) cells. Presence of DC-LAMP^+^ mature DCs within TLSs, but not CD68^+^ macrophages. Inset shows hematoxylin and eosin stain of same section. Adjacent small panels show original images of same section stained using chromogenic immunohistochemistry detection with several immune markers and overlay of pseudo-colored images obtained using ImageJ. Inset in pseudo-color images shows a zoomed-in view. (*F*) Staining for GrB CD8 T cells in a patient with TLSs (upper panels), a patient with lymphoid aggregate (TLS^–^ patient, middle panels), and a patient without TLSs (bottom panels). The first panel shows immunofluorescence staining for TLSs. The second panel shows immunohistochemistry for CD8 of sequential section. The third panel shows same section, stripped and reprobed for granzyme B (arrowheads) with inset showing magnified area. The fourth panel shows correspondent pseudo-color immunofluorescence image. (*G*) Difference of CD8^+^ CD3^+^ T cell infiltration in TLS^+^ vs TLS^–^ KPC tumors. Two-samples Kolmogorov-Smirnov test: ∗*P =* .0286. (*H*) Plot showing the significant correlation of CD8^+^ T cells with TLS density in KPC mice. Empty circles represent TLS^+^ KPC tumors, bold circles represent TLS^–^ KPC tumors. Empty circles represent TLS^+^ PDAC tumors, bold circles represent TLS^–^ PDAC tumors. Two-sample Kolmogorov-Smirnov test: (*B*) ∗*P =* .0014 and (*G*) Spearman r = 0.76, *P =* .04. Scale bar: (*A–D*) 50 μm, (*E*) 200 μm, (*F*) 100 μm.

**Figure 4:**
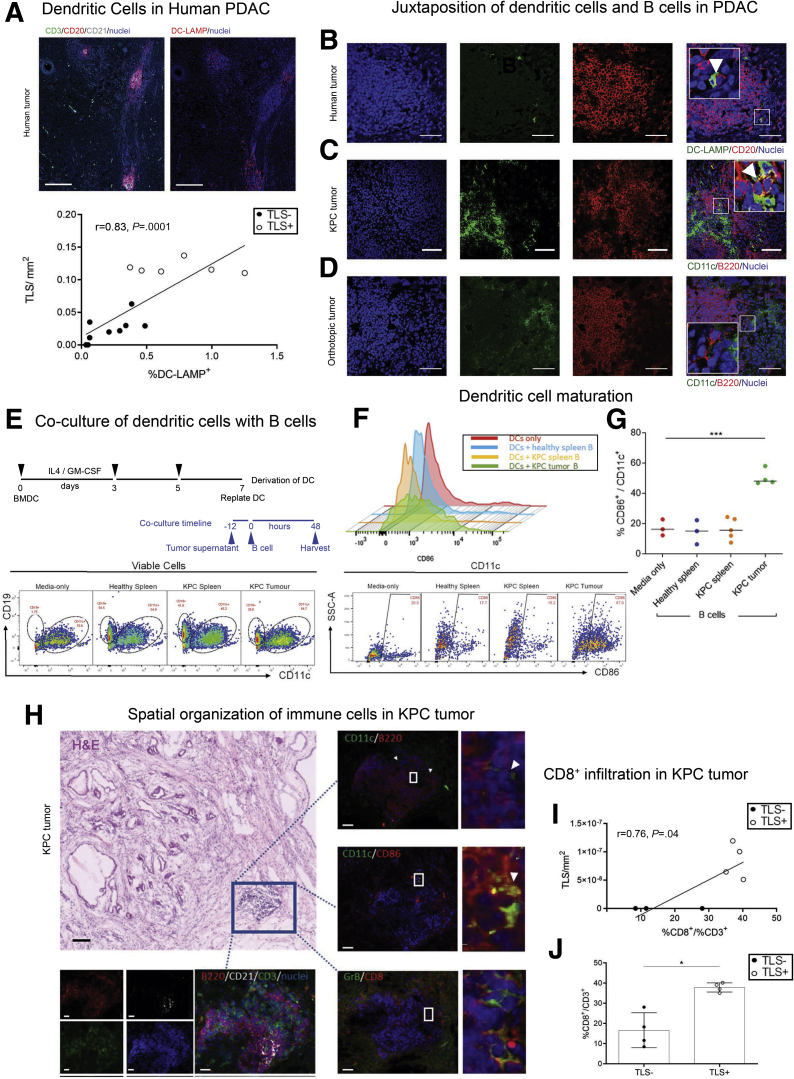
**TLS-associated B cells provide significant help in priming the antitumor response.** (*A*) IF staining for TLSs (upper left panel) and DC-LAMP (upper right panel) of sequential human PDAC sections. Plot (bottom panel) showing the significant correlation of DC-LAMP^+^ cells with TLS density. Spearman r = 0.83, *P =* .0001. (*B*) Dendritic cells (DC-Lamp^+^) colocalization with B cells (CD20^+^) in human TLSs. (*C*) Dendritic cells (CD11c^+^) colocalization with B cells (B220^+^) in KPC TLSs. (*D*) Absence of dendritic cells (CD11c^+^) colocalization with B cells (B220^+^) in orthotopic TLSs. (*E*) Timeline and gating strategy of in vitro co-culture of BMDCs and B cells from healthy spleen, KPC spleen, and KPC tumors to assess dendritic cell activation by flow cytometric analysis. (*F*) Representative overlay histograms and representative dot plots of flow cytometry analysis for CD86 expression on dendritic cells after 48 hours of co-culture with B cells from KPC spleen and tumor and healthy spleen B cells. No co-culture (media only) as negative control. The FMO (fluorescence minus 1) for CD86 was used to define the gate. (*G*) Quantification of expression of CD86 on dendritic cells after 48 of co-culture, and no co-culture (media only) as negative control. One-way analysis of variance and Bonferroni's posttest: ∗∗∗*P* < .001. (*H*) KPC hematoxylin and eosin section demonstrating relationship of TLSs (box) to tumor tissue, confirmed by staining on sequential sections for CD3^+^ (green)/B220^+^ (red)/CD21^+^ (white) TLSs (lower left panel), and juxtaposition of CD11c^+^ dendritic (green)/B220^+^ B (red) cells (upper right panel), costaining demonstrating CD11c^+^ (green)/CD86^+^ (red) dendritic cells (middle right panel), and granzyme B^+^ (green)/CD8^+^ (red) T cells (bottom right panel) with respective boxes highlighted on the right and arrowheads pointing to appropriate cell types. (*I*) Plot showing the significant correlation of CD8^+^ T cells with TLS density in KPC mice. Empty circles represent TLS^+^ KPC tumors, bold circles represent TLS^–^ KPC tumors. Spearman r = 0.76, *P =* .04. (*J*) Difference of CD8^+^ CD3^+^ T cell infiltration in TLS^+^ vs TLS^–^ KPC tumors. Two-samples Kolmogorov-Smirnov test: ∗*P =* .0286. Empty circles represent TLS^+^ PDAC patients, bold circles represent TLS^–^ PDAC patients. Scale bars: (*A*) 250 μm, (*B–D*) 50 μm; (*H*) 20 μm for IF images, 100 μm for hematoxylin and eosin.

We could detect germinal centers in human PDAC TLSs, as indicated by expression of the nuclear protein B cell lymphoma 6 (BCL6) (and AID [activation-induced deaminase]), as well as in KPC tumors (expressing the GL7 antigen) but not in orthotopic PDAC tumors, suggesting presence of mature, functionally active TLSs only in human and KPC PDAC tumors (Figure 3*A–D*). Focal expression of BCL6 and AID in mature TLSs is suggestive of antitumor B cell selection and expansion (Figure 3*A* and *B*), and presence of CD8^+^ T cells is indicative of a cytotoxic immune response, as suggested by expression of T cell intracellular antigen and granzyme B, and exclusion of T regulatory (Foxp3^+^) cells from the TLS core (Figure 3*E–H*). Moreover, CD68^+^ cells are excluded from TLSs, while DC-Lamp^+^ mature dendritic cells home selectively in TLSs in PDAC (Figure 3*E* and 4*A*), supporting the involvement of TLSs in the promotion of a protective adaptive immunity, as shown in patients with early-stage non-small cell lung cancer.^19^^,^^20^

### TLSs Are Sites for Antigen Presentation

In addition, anatomically TLSs may provide a site for antigen presentation. Dendritic cells are the most efficient antigen-presenting cells but require maturation and activation.^21^ We could demonstrate a clear juxtaposition of dendritic cells with B cells within TLSs in human and KPC tumors, suggesting crosstalk within TLSs, not seen in orthotopic PDAC tumors (Figure 4*B–D*). We postulated that, in this spatial organization, B cells may facilitate DC maturation.^22^ In order to explore this, we isolated B cells from KPC tumors (where they are almost exclusively present in the TLS, and rarely found as single cell infiltrate) and co-cultured them with bone marrow–derived dendritic cells (BMDCs) isolated from syngeneic healthy mice, in the presence or absence of KPC tumor cell-conditioned media (Figure 4*E*). Simultaneously, we used B cells isolated from either healthy or KPC spleens (secondary lymphoid organs [SLOs]) as controls (Figure 4*E–G*). Within 48 hours of co-culture, intratumoral B cells caused a 3-fold upregulation of costimulatory molecule CD86 on DCs (as well as larger cell size [data not shown]), to a degree similar to lipopolysaccharide stimulation, while B cells isolated from either healthy or KPC spleens did not cause DC activation (Figure 4*F* and *G*). Furthermore, immunostaining of sequential KPC sections showed expression of CD86 by CD11c^+^ dendritic cells and presence of granzyme B^+^ CD8^+^ T cells within the TLS (Figure 4*H–J*). Taken together, these data indicate that B cells within TLSs acquire immunomodulatory ability, resulting in induction of upregulation of immunostimulatory molecules on dendritic cells. These aspects are suggestive of a role for B cells within TLSs, where dendritic and other important cells can be conveniently accessed, in order to mount a de novo antitumor immune response.^14^^,^^23^

### Coinjection of CXCL13 and CCL21 Into Orthotopic Tumors Recruits B and T Cells and Facilitates TLS Formation

**Figure 5:**
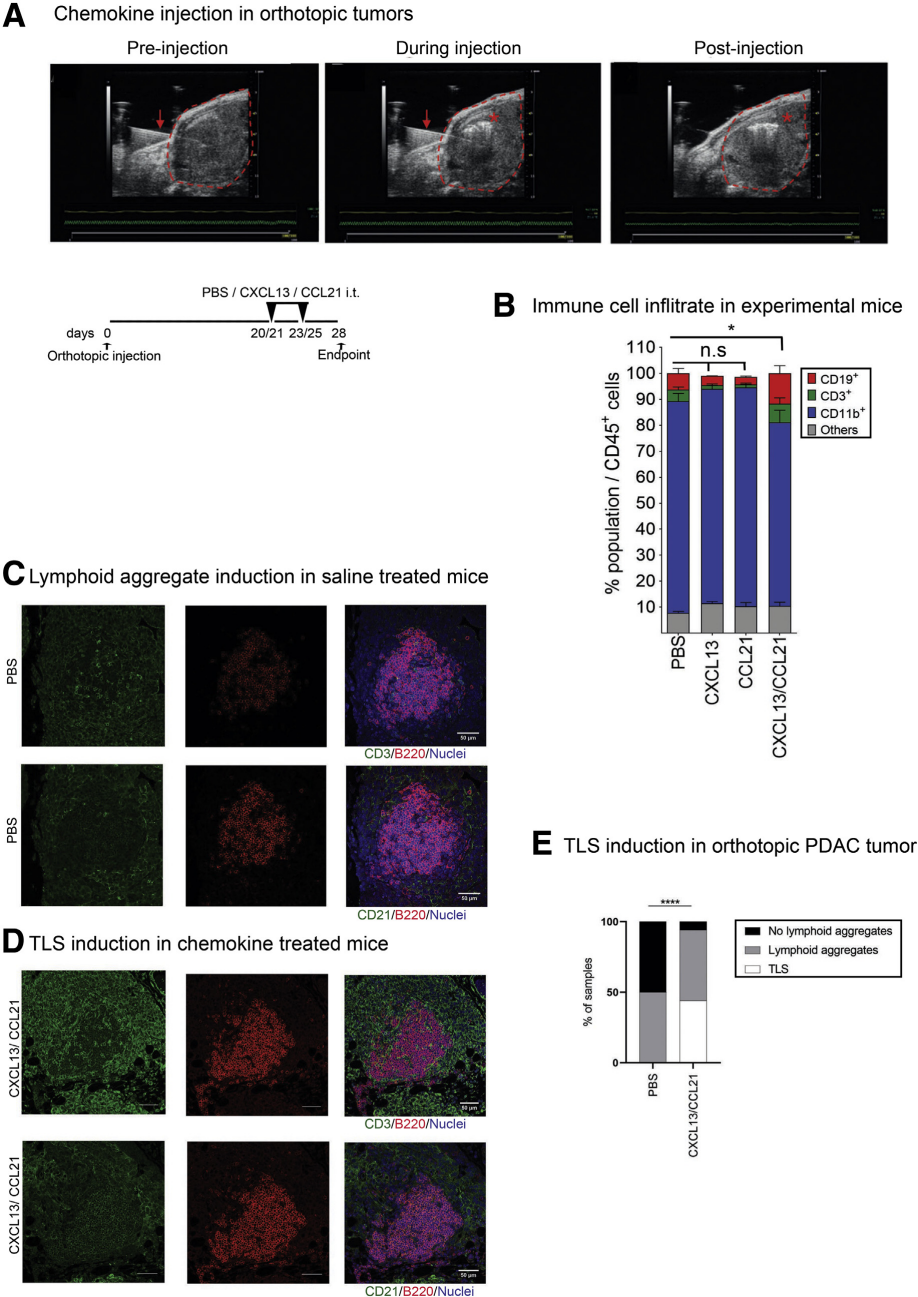
**Artificial induction of TLSs in orthotopic model of PDAC.** (*A*) Ultrasound images of the pancreatic tumor of orthotopic mice preinjection (left panel), during injection (middle panel), and postinjection (right panel) and schema of intratumoral injection of lymphoid chemokines CXCL13 and CCL21 after development of orthotopic PDAC tumor. Red arrow: needle trajectory; red dotted line: tumor; red asterisk: spread of solution. Schema of experiment. (*B*) Proportion of CD3^+^, CD19^+^ CD11b^+^ and other immune cells out of total CD45^+^ cells after intratumor lymphoid chemokine injection as assessed by flow cytometric analysis (PBS n = 12, CXCL13/CCL21 n = 16, CXCL13 n = 4, CCL21 n = 4). (*C*) Representative immunofluorescence staining on sequential sections of lymphoid aggregates as detected by the presence of B cells (B220), the presence of T cells (CD3) (upper panels), and the near absence of FDCs (CD21) (lower panels) with DAPI staining nuclei in PBS (vehicle control) injected mice. (*D*) Representative immunofluorescence images of TLSs as detected by the presence of B cells (B220), T cells (CD3) (upper panels), and well-formed network of FDCs (CD21) (lower panels) with DAPI staining nuclei in dual chemokine-injected mice, showing mature TLSs. (*E*) Lymphoid aggregate and TLSs induction in CXCL13/CCL21 intratumoral coinjected mice. (PBS n = 12, CXCL13/CCL21 n = 16). (*B*) Kruskal-Wallis and (*E*) chi-square test. ∗*P <* .05, ∗∗∗∗*P <* .0001. Scale bar: 50 μm. n.s, not significant.

Consequently, we wanted to assess if TLSs could play an antitumor role if induced into orthotopic PDAC tumors.^9^^,^^23^, ^24^, ^25^, ^26^, ^27^, ^28^ The desmoplastic stroma of human PDAC secretes a number of chemokines, which may facilitate a differential immune cell infiltrate,^23^ a feature characteristically missing in orthotopic PDAC models.^24^ This key difference may, at least in part, account for the absence of TLSs in this model; however, TLSs are seen when cancer cells are orthotopically coinjected with murine pancreatic stellate cells (data not shown). Chemokines CXCL13 (Figure 1*B*) and CCL21^9^ are present in human PDAC TLSs. CXCL13 selectively recruits B cells^25^ and CXCL13 inhibition reduced B cell infiltration in orthotopic tumors.^26^ CCL21 recruits naïve T cells, natural killer cells, and dendritic cells.^27^ Hence, we administered CXCL13 and CCL21 individually or concurrently intratumorally as a strategy to induce TLS formation in the orthotopic PDAC model (Figure 5*A*). As anticipated, coadministration of CXCL13/CCL21 was able to induce a significant increase in lymphoid immune infiltrate, and was associated with a significant reduction of myeloid cells proportion (Figure 5*B*).

**Figure 6:**
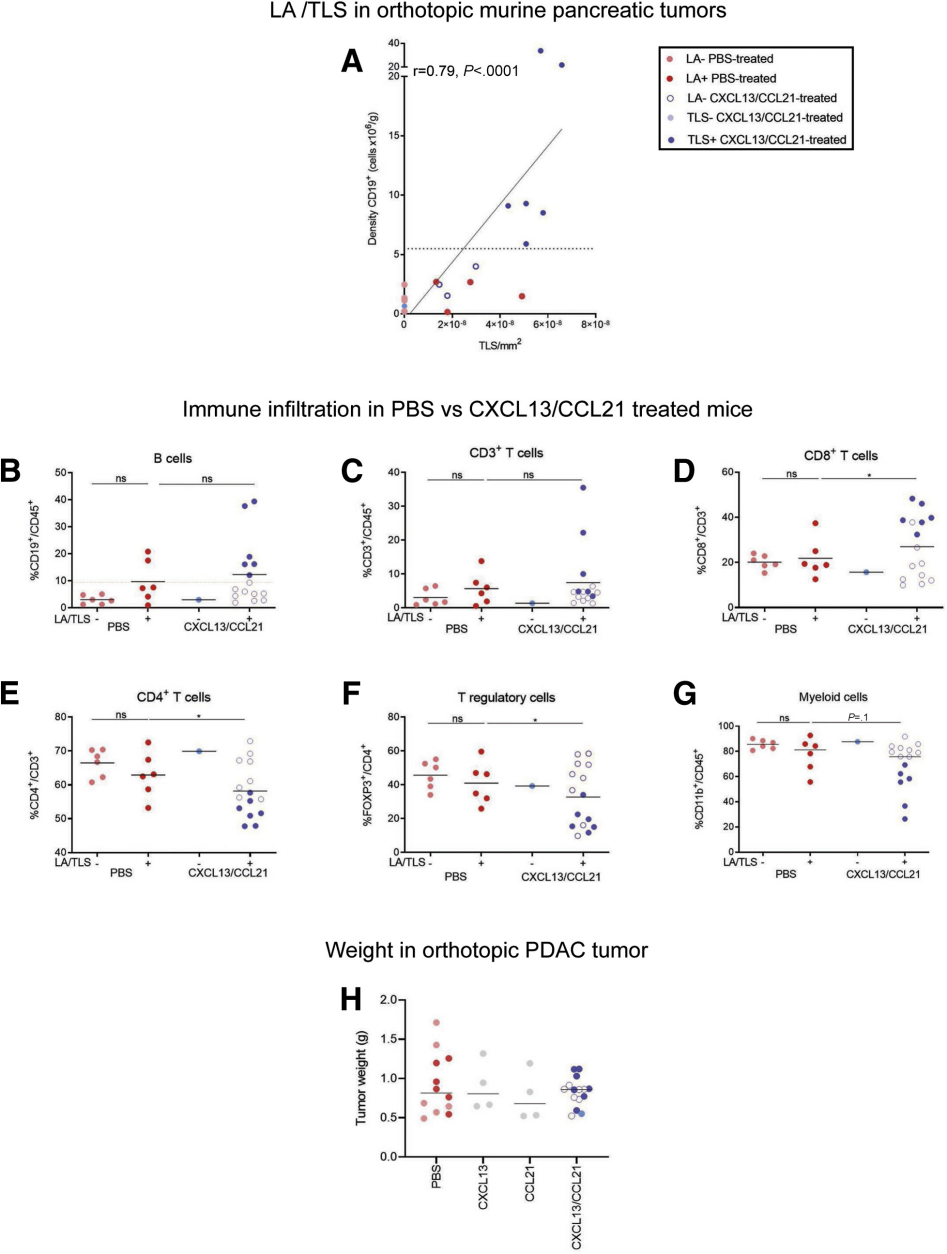
**Coinjection of CXCL13 and CCL21 into orthotopic tumors.** (*A*) Correlation of CD19^+^ B cell density with TLS density in orthotopic pancreatic tumors. The dotted line represents the cutoff of minimal B cell density needed to induce lymphoneogenesis. Pink circles represent lymphoid aggregate (LA)^–^ PBS-treated mice, red circles represent LA^+^ PBS-treated mice, blue circles represent TLS^+^ CXCL13/CCL21-treated mice, empty blue circles represent stress-induced LA^+^ CXCL13/CCL21-treated mice. Spearman r = 0.79, *P* < .0001. (*B–G*) Flow cytometric analysis, in LA^+/–^ tumors from PBS-treated mice (n = 12) and TLS^+/–^ tumors from CXCL13/CCL21-treated mice (n = 16), of (*B*) CD19^+^ B cells out of CD45^+^ cells, (*C*) CD3^+^ T cells out of CD45^+^ cells, (*D*) CD4^+^ helper T cells out of CD3^+^ T cells, (*E*) CD8^+^ cytotoxic T cells out of CD3^+^ T cells, (*F*) FOXP3^+^ regulatory T cells out of CD4^+^CD3^+^ T cells, and (*G*) CD11b^+^ myeloid cells out of CD45^+^ cells. The mean percentage of CD19^+^ cells out of CD45^+^ cells within the PBS-treated group used to identify stress-induced lymphoid aggregates from potential chemokine-induced TLSs (dotted line in A). (*H*) Tumor weight in CXCL13/CCL21 intratumoral single or coinjected compared with PBS-treated mice. Each data point represents 1 mouse. Empty circles in CXCL13/CCL21 treated mice represent stress-induced lymphoid aggregates. Two-sample Kolmogorov-Smirnov test. ∗*P <* .05. ns, not significant.

The process of injection induced focal small lymphoid aggregate formation in the phosphate-buffered saline (PBS)–injected mice (control group) (Figure 5*C–E*), an unexpected feature that was not accounted for during experimental design. Though these did not appear to fulfill all the immunophenotypic criteria for TLSs (dense, compact B and T cell aggregate with FDC network, and critical mass of B cells) (Figures 5*C* and *D* and 6*A*), we assessed this control group in more detail with regard to lymphoid subset infiltrate. We could not detect any difference in the immune infiltrate based on the presence or absence of lymphoid aggregates, in PBS-treated mice (Figure 6*B–G*), suggesting that this spatial aggregation of the lymphoid cells (lymphoid aggregate) in the control group was a focal reaction to injection-induced trauma, which was also observed in chemokine-treated mice. Importantly, these lymphoid aggregates were immunologically distinct from the fully formed TLSs seen in chemokine-treated mice (Figure 6*B–G*). In chemokine-treated mice, high B cell infiltrate was associated with TLS formation (Figures 5*D* and 6*A*).

Alongside the immunophenotypic criteria for TLS identification, the mean value of %CD19^+^/CD45^+^ of the PBS-injected mice with lymphoid aggregate was used to objectively discriminate between stress-induced lymphoid aggregates and potentially chemokine (CXCL13/CCL21)-induced TLSs within the chemokine-treated mice (Figure 6*B–G*). This led to a clear discrimination of the chemokine treated mice into 2 groups: those with TLSs (46%) and those without (54%) (Figures 5*E* and 6).

**Figure 7:**
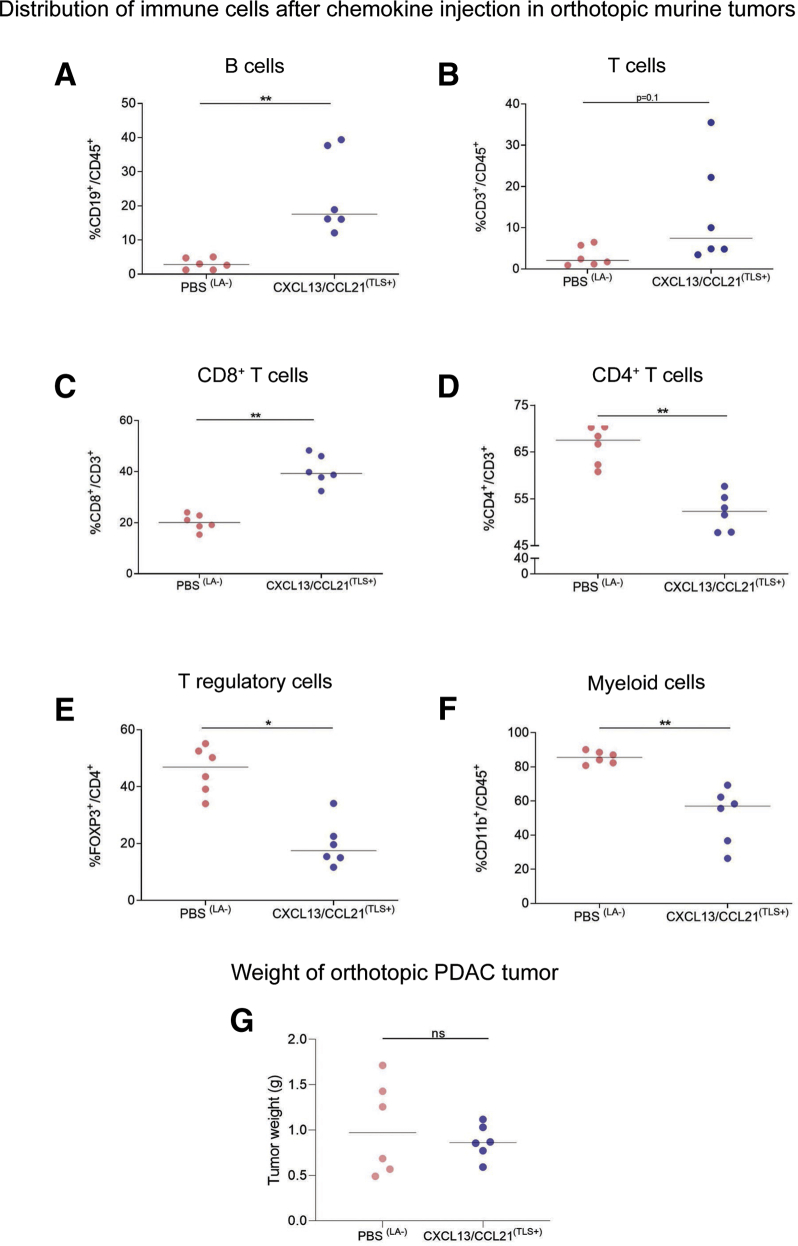
**Differential immune cell infiltration after chemokine injection in orthotopic murine tumors.** Flow cytometric analysis, in lymphoid aggregate negative tumors from PBS-treated (n = 6) and TLS^+^ CXCL13/CCL21-treated (n = 6) mice, of (*A*) CD19^+^ B cells out of CD45^+^ cells, (*B*) CD3^+^ T cells out of CD45^+^ cells, (*C*) CD8^+^ cytotoxic T cells out of CD3^+^ T cells, (*D*) CD4^+^ helper T cells out of CD3^+^ T cells, (*E*) FOXP3^+^ regulatory T cells out of CD4+CD3^+^ T cells, (*F*) CD11b^+^ myeloid cells out of CD45^+^ cells, and (*G*) tumor weight in LA^–^tumors from PBS-treated (n = 6) and TLS^+^ CXCL13/CCL21–treated (n = 6) mice. Each data point represents 1 mouse. Two-sample Kolmogorov-Smirnov test: ∗*P <* .05 and ∗∗*P* < .01. ns, not significant.

We also demonstrate that the immune infiltrate within PBS-treated mice (with lymphoid aggregates) differed from CXCL13/CCL21-treated mice (with chemokine-induced TLSs and lymphoid aggregates). While there was no alteration in overall T cell infiltrate, there was a significant reduction in CD4^+^, FoxP3^+^, and CD11b^+^ cells and an increase in CD8^+^ cells in tumors after chemokine injection compared with the control group. These changes were most pronounced in mice presenting a higher B cell fraction (Figure 6*B–G*). Consequently, a subgroup comparison was conducted to exclude the artifact introduced by lymphoid aggregate formation at injection sites using the immunophenotypic criteria and the %CD19^+^/CD45^+^ threshold to compare the control group with TLS-bearing, chemokine-injected group (Figure 7). In this post hoc subgroup analysis (PBS^LA–^ vs CXCL13/CCL21^TLS+^), we observed that chemokine injection promoted CD8^+^ T cell infiltration and reduced CD4^+^ T cell infiltration, despite no alteration in total T cell (CD3^+^/CD45^+^) proportion. In particular, chemokines facilitated reduction in the FoxP3^+^ subpopulation (akin to FoxP3^+^ exclusion in human TLSs) (Figure 3*E*) and the CD11b^+^ fraction within the chemokine-injected TLS-bearing tumors (Figure 7*A–F*). Certainly, in this pilot experiment, we could demonstrate that formation of chemokine-induced TLSs is associated in changes in immune microenvironment of the tumor. Despite that the mere induction of TLSs had no impact on tumor growth, we cannot exclude this is due to the short time frame of tumor development (Figure 7*G*).

### Coadministration of Systemic Chemotherapy and Lymphoid Chemokines Leads to Significant Tumor Reduction

**Figure 8:**
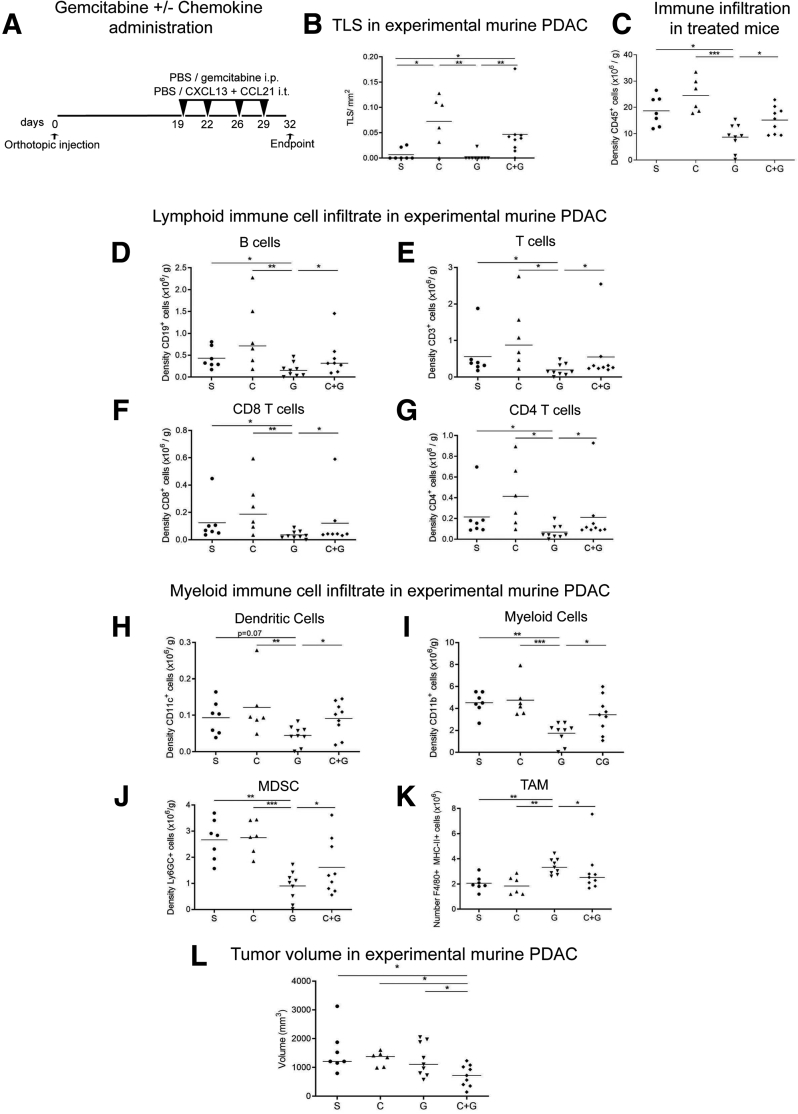
**Combination of chemotherapy and immune activation within intratumoral TLSs is necessary for antitumor activity.** (*A*) Schema of short-term administration of gemcitabine in combination or not with intratumoral injection of lymphoid chemokines CXCL13 and CCL21 in orthotopic mice. (*B*) TLS density after coinjection of gemcitabine and chemokine (C+G) compared with appropriate controls (PBS [S], chemokine [C], or gemcitabine [G] alone. (*C*) Flow cytometric analysis of CD45^+^ immune cells in PBS-treated (S) (n = 7) gemcitabine-treated (G) (n = 9) mice, chemokine-treated (C) (n = 6), and C+G-treated (n = 8) mice. Flow cytometric analysis of lymphoid and myeloid immune cells per gram of tumor tissue after chemokine and/or chemotherapy injection. (*D*) B cells (CD19^+^), (*E*) CD3^+^ T cells, (*F*) CD8^+^ T cells, (*G*) CD4^+^ T cells, (*H*) dendritic cells (CD11c^+^), (*I*) myeloid cells (CD11b^+^), (*J*) MDSC Ly6GC^+^ subset, (*K*) macrophages (F4/80^+^, MHC-II^+^). (*L*) Tumor volume in C+G-coinjected mice and appropriate controls (chemokines [C], or gemcitabine [G] alone). Each data point represents 1 mouse (S n = 7, C n = 6, G n = 9, C+G n = 9). Kolmogorov-Smirnov test. ∗*P <* .05, ∗∗*P <* .01, ∗∗∗*P <* .001.

Because the injection of CXCL13 and CCL21 indicated a potential antitumor immune activity in orthotopic tumors, we explored whether coadministration of chemotherapy to induce tumor cell death, and perhaps release of tumor antigens,^29^ could impact tumor dynamics (Figure 8*A* and *B*). We chose gemcitabine as a chemotherapeutic agent, as it is commonly used for pancreatic cancer patients.^1^ Simultaneous chemotherapy and chemokine administration showed interesting changes compared with single administration of either agents. It is well known that chemotherapeutic treatments, such as gemcitabine, are associated with a significant impact on the immune system, which, in turn, may contribute to the limited efficacy in pancreatic cancer treatment.^30^ In our model, treatment with gemcitabine caused a global reduction of immune cell infiltrate compared with vehicle-treated mice, as measured in number of cells per gram tumor tissue (Figure 8*C*), akin to the well-recognized effect on circulating white cells after gemcitabine administration in human.

In contrast, chemokine (CXCL13/CCL21) injection either alone or alongside gemcitabine demonstrated a significant improvement in immune infiltration compared with gemcitabine alone, apart from TAM (defined as percentage of F4/80^+^MHCII^+^/CD45^+^ cells), which were reduced (Figure 8*D–K*), indicating a possible immunostimulatory microenvironment induced by chemokine injection. Surprisingly, the combination of gemcitabine and chemokine injection resulted in smaller tumors (Figure 8*L*), suggesting that combining chemotherapy with appropriate immunotherapy to induce an immunostimulatory microenvironment could be used to tailor more personalized treatment.

**Figure 9:**
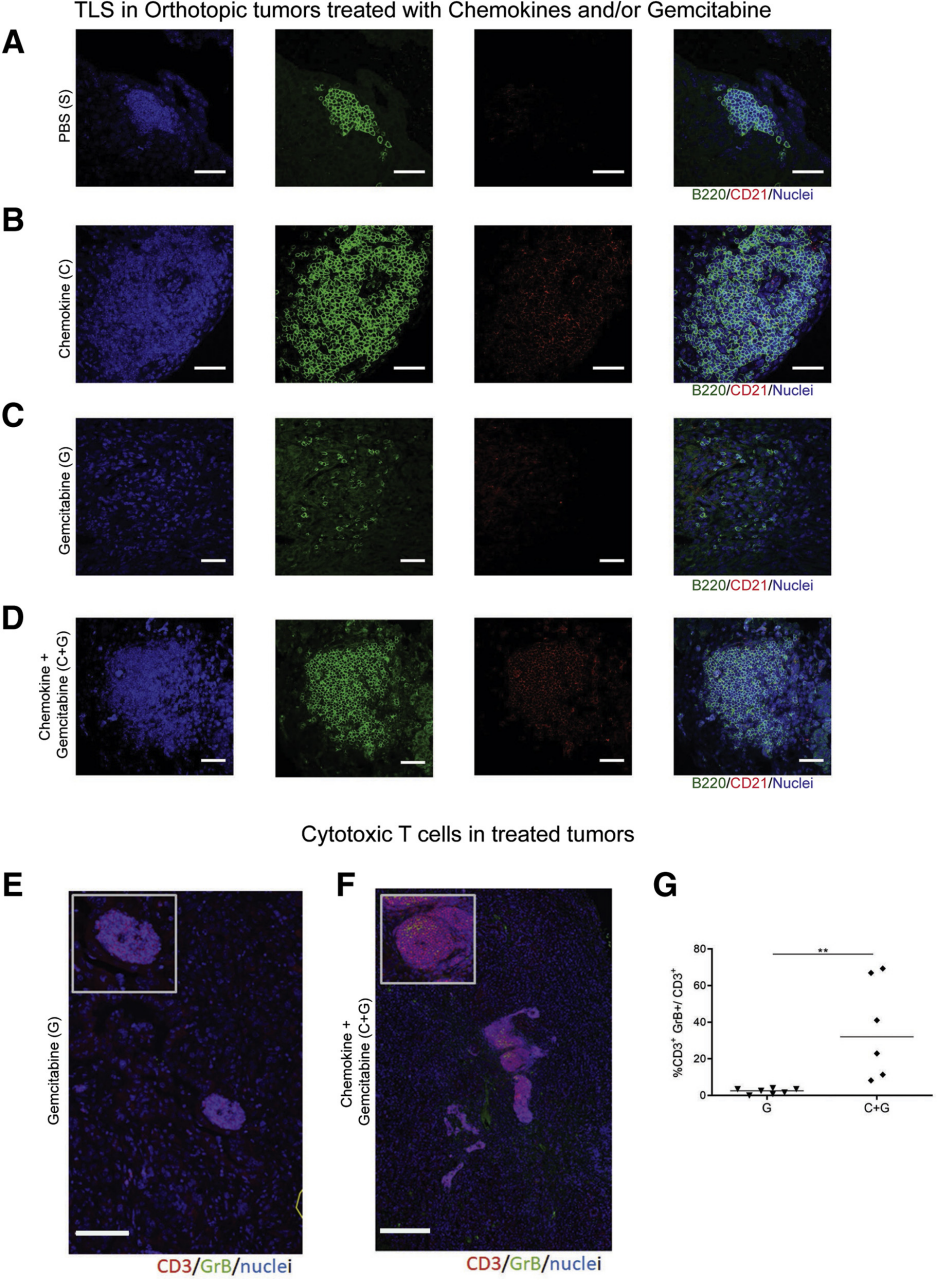
**Coinjection of CXCL13 and CCL21 into orthotopic tumors, and administration of intraperitoneal gemcitabine.** (*A–D*) Representative immunofluorescence images of aggregates or TLSs as detected by the presence of B220 (green), and CD21 (red) with DAPI (blue) staining nuclei in (*A*) PBS-treated, (*B*) chemokine-treated, (*C*) gemcitabine-treated, (*D*) and C+G-treated mice. (*E*, *F*) IF staining of gemcitabine- and C+G-treated mice for granzyme B (green) and CD3 (red) T cells. Insets represent a zoomed-in view of the aggregate. (*G*) Quantification of the granzyme B^+^ T cells in gemcitabine alone (G) and in combination with C+G-treated mice. Two-samples Kolmogorov-Smirnov test: ∗∗*P <* .01. Scale bar, (*A–D*) 50 μm, (*E*, *F*) 250μm .

We noted a substantial immune cell infiltrate and sustained TLS formation following chemokine injections, both with or without chemotherapy, as evidenced by colocalization of B220^+^ (B cells) and CD21^+^ (FDCs) cells in clusters, within these tumors (Figure 8*B* and 9*A–D*). Furthermore, we observed an increase of CD8^+^ GrB^+^ T cell infiltration in chemokine and gemcitabine–treated mice, compared with gemcitabine alone, and a localization of these cytotoxic T cells within aggregates (Figure 9*E–G*).

**Figure 10:**
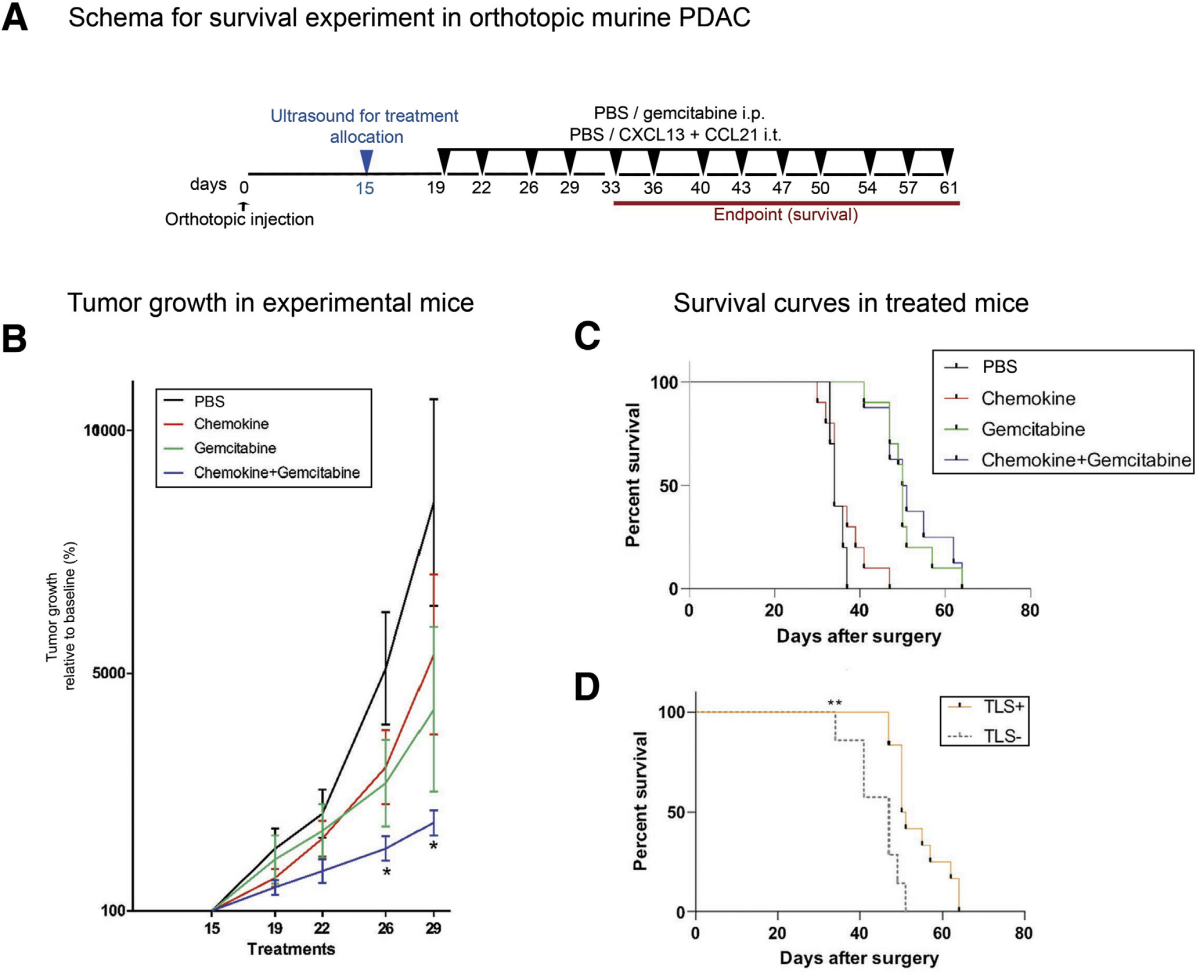
**Combination of chemotherapy and immune activation within intratumoral TLSs improves survival in the orthotopic model of PDAC.** (*A*) Schema of administration of gemcitabine in combination with intratumoral injection of lymphoid chemokines CXCL13 and CCL21 in orthotopic mice (C+G) (n = 10) for the lifespan of the mice. Controls were represented by intraperitoneal and intratumoral injections of PBS for the control group (S) (n = 10), intraperitoneal PBS injection and intratumoral chemokines injection for the chemokine-alone group (C) (n = 10), and intraperitoneal gemcitabine injections and intratumoral PBS injections for the gemcitabine group (G) (n = 10). (*B*) Tumor volume change with baseline at day 15 (100%) up to day 29 when all mice were available for measurements (before endpoint). (*C*) Kaplan-Meier survival curve for the 4 treatment arms. (*C*) Kaplan-Meier survival curve for subgroup of mice treated with chemotherapy with or without chemokines analyzed for presence or absence of TLSs. (*B*) Linear mixed-effect models, with Dunnett’s adjustment for multiple comparison, (*C*, *D*) Log-rank (Mantel-Cox) test. ∗*P <* .05; ∗∗*P <* .01; ∗∗∗∗*P <* .0001.

Next, we performed a longer experiment (Figure 10*A*) in order to assess survival. As expected, the combination treatment resulted in reduction in tumor volume up to 29 days, when all mice were available for measurements (Figure 10*B*). Overall, chemokine addition did not improve survival compared with gemcitabine alone (Figure 10*C*). However, among mice receiving gemcitabine with or without chemokine, we could demonstrate a survival advantage in mice with TLS-containing tumors (Figure 10*D*).

## Discussion

In this study, we demonstrate that when B and T cells can arrange in organized structures such as TLSs, they contribute to reducing tumor growth in the presence of chemotherapy. Our detailed in vivo experimental approach gives credence to the observations that presence of TLSs has a favorable prognostic impact in patients with PDAC.^9^ While the exact mechanism of this antitumor activity within TLSs remains to be fully elucidated, we demonstrate, as a first step, that juxtaposed localization of B and dendritic cells in an organized structure, such as TLSs, may allow for a better intercellular communication, which may potentiate the adaptive antitumor response. This is in contrast to the existing paradigm that tumor infiltration is based on the activation of lymphocyte precursors at extra-tumoral sites, such as the nearest SLO (lymph nodes or spleen), followed by migration of these activated lymphocytes into the circulation to reach tumor sites.^31^ TLSs may represent a more efficient strategy for B and T cell priming, obviating the need to migrate to the draining lymph node to present the loaded antigen, although the 2 paths may not be mutually exclusive. Once active, because effector cells are already within the tumor vicinity, they could potentially exert their function locally with no need to cross any micro-environmental barrier.

TLSs, as demonstrated herein, occur only in a fraction of patients and share cellular composition and compartmentalization in B cell– and T cell–rich zones, chemokines, vasculature, and function with SLOs.^8^^,^^12^^,^^13^^,^^32^, ^33^, ^34^, ^35^, ^36^ Their presence consistently correlates with favorable prognosis.

Recently, not only TLS density, but also TLS maturation, called TLS immunoscore, have been shown to prognosticate the risk of disease recurrence in untreated nonmetastatic colorectal cancer.^16^ Mature TLSs are the ultimate stage of TLS formation, in which developed follicle-like structures show the presence of germinal centers.^16^ Furthermore, the immune rich with TLS subtype of PDAC has been described and it exhibits perturbations in genes involved in DNA repair^6^^,^^7^; this is known to increase the number of tumor neoantigens, which can provoke an immune response. The in situ presence of TLSs may help a faster immune humoral and adaptive response to a rapidly evolving immunogenic landscape.

Mature TLSs in PDAC before any immune intervention may be used as a biomarker to define inclusion criteria of patients in immunotherapy protocols, with the aim to boost the ongoing antitumor immune response. Furthermore, our study shows that the lymphoid chemokines CXCL13 and CCL21, when used in combination with cytotoxic chemotherapy, represent a viable therapeutic strategy for the modulation of TLSs, promoting a stable and long-lasting antitumor immune response, which could lead to better clinical outcome in patients. Dissecting the dynamics of human TLSs would, for example, enable vaccines such as granulocyte-macrophage colony-stimulating factor–secreting vaccines to be harnessed to full therapeutic potential.^8^ It is known that gemcitabine, gemcitabine/nab-Paclitaxel and FOLFIRINOX regimens are associated with Grade III hematologic adverse effects.^37^^,^^38^ We show that the resultant paucity of leukocytes may be overcome by administration of lymphoid chemokines, which may restore the lymphoid immune infiltration in PDAC Tumor Micro-environment (TME). A combinatorial therapeutic approach that may potentiate formation of active TLSs, and ablation of the immune suppression (eg, suppressive cytokine inhibitors, such as galunisertib)^39^ could further enhance the efficacy of chemotherapy.

The variability of de novo TLS presence in human PDAC can be hypothesized to be due to tumoral^4^^,^^6^ and stromal heterogeneity.^40^ Intriguingly, recent data from our laboratory suggest that cancer-associated fibroblasts demonstrate intertumoral and intratumoral heterogeneity and are highly plastic with at least 4 well-defined subtypes.^41^ Differential expression of chemokines between these subtypes may facilitate spatial immune segregation within a single tumor, with the potential to play a crucial role in intrastromal (cancer-associated fibroblast–immune cells) crosstalk, an aspect that can be targeted.^42^ We hypothesize that a better understanding of the temporal development of TLSs along with activation of T and B cells^43^^,^^44^ leading to their antitumoral functions, may provide therapeutic insight into the treatment of PDAC.

## Materials and Methods

### Study Approval

Human PDAC TMAs were constructed as previously reported (City and East London Research Ethics Committee 07/H0705/87).^23^ Briefly, 6 cores (1-mm diameter each) per patient (n = 56) were taken from the tumor and stroma area, and only those patients with at least 3 cores (at least 1 each from tumor or stroma) were included for TLS analysis. Correspondent full sections of 14 patients were used to validate TMA findings. Another set of tissue samples (n = 17) were provided as full sections by the Barts Pancreatic Tissue Bank (London, United Kingdom) in accordance with the regulations of the tissue bank^45^ and approvals (Barts Pancreatic Tissue Bank approval 2020/05/QM/HK/E/FFPE_Tissue). Animal procedures were carried out in accordance with the UK Home Office Animal and Scientific Procedures Act 1986 and the European Directive 2010/63/EU, under the project licenses 70/7411, 70/7449, and PBE3719B3.

### Immunofluorescent Staining of Human Samples

Whole sections and TMAs were used for immunostaining. Sections were deparaffinized and rehydrated before undergoing antigen-retrieval (citrate buffer, pH 6), blocking (2% bovine serum albumin [#A8022; Sigma-Aldrich, St Louis, MO]), 0.02% fish skin gelatin (#G-7765 [Sigma-Aldrich] or #X0909 [Dako, Glostrup, Denmark]), and washing (0.01% Tween-20 in PBS [PBS-T] or in Tris-buffered saline) steps. Primary antibodies (Table 1) diluted in blocking buffer or antibody diluent (#S3022 [Dako] or #936B-08 [Sigma-Aldrich]) were applied and incubated overnight (4°C) or 30 minutes (room temperature [RT]) before secondary antibody incubation (1 hour, RT) (Table 2). When tyramide was used to detect primary antibody binding, a second antigen retrieval process was used before staining with 2 further primary and secondary antibody combinations. Sections were counterstained with DAPI (#62248; Life Technologies, Carlsbad, CA) and slides mounted using ProLong Gold Antifade mounting solution containing DAPI (#P-36931; Life Technologies). Imaging was performed using the confocal microscopes LSM510/710, Axioscan.Z1 (Zeiss, Oberkochen, Germany) and NanoZoomer S60 (Hamamatsu Photonics, Hamamatsu, Japan).

**Table 1:** Primary Antibodies for Immunofluorescence

Species Raised in	Antibody	Fluorochrome	Clone	Company	Catalog Number
Rat	B220	Purified	RA3-6B2	BD Biosciences	550286
Rat	B220	AF647	RA3-6B2	BioLegend	103226
Rat	B220	FITC	RA3-6B2	eBioscience	11-0452-85
Rabbit	BCL6	Purified	Polyclonal	Abcam	ab220092
Armenian hamster	CD11c	biotinylated	N418	eBioscience	13-0114-85
Mouse	CD20	Purified	L26	Dako	M0755
Rabbit	CD21	Purified	EP3093	Abcam	75985
Rat	CD21/CD35	PE	7E9	BioLegend	123409
Rabbit	CD3	Purified	Polyclonal	Abcam	5690
Rat	CD3	APC	17A2	BioLegend	100236
Rat	CD3	FITC	17A2	BioLegend	100203
Mouse	CD8	Purified	C8/144B	Dako	M7103
Rat	CD86	Purified	AP-MAB0803	Novus	NBP2-12182
Rat	CD8a	PE	53-6.7	BD Pharmingen	553032
Rabbit	DC-LAMP	Purified	Polyclonal	Protein Atlas	HPA051467
Rat	FDC-M1	Purified	Rat IgG2c	BD Biosciences	551320
Rabbit	GL-7	FITC	GL7	BioLegend	144604
Mouse	Granzyme B	Purified	GrB-7	Dako	M7235
Rat	PNAd	Purified	MECA-79	BD Pharmingen	553863
Mouse	IgG	Purified		Dako	X0931
Rabbit	IgG	Purified		Abcam	2747
Rat	IgG	Purified		Abcam	37361

**Table 2:** Secondary Antibodies for Immunofluorescence

Species Raised in	Reactivity	Fluorochrome	Company	Catalog Number
Goat	Rabbit IgG	Alexa Fluor 488	Invitrogen	A11034
Streptavidin	Biotin	Alexa Fluor 488	Thermo Fisher Scientific	S11223
Tyramide reagent		Alexa Fluor 488	Invitrogen	B40953
Goat	Hamster	Alexa Fluor 488	Invitrogen	A21110
Goat	Mouse	Alexa Fluor 488	Invitrogen	A11029
Goat	Mouse IgG	Alexa Fluor 546	Invitrogen	A11030
Goat	Rat	Alexa Fluor 546	Invitrogen	A11081
Goat	Rabbit	Alexa Fluor 546	Invitrogen	A11035
Goat	Rat IgG	Alexa Fluor 633	Invitrogen	A21094
Goat	Mouse	Alexa Fluor 633	Molecular Probes	A-21052
Donkey	Rabbit	Alexa Fluor 647	Invitrogen	A32795

### Stripping and Reprobing Immunohistochemistry Staining of Human Samples

The stripping and reprobing protocol is a modification of a previously described immunohistochemistry protocol.^46^ Briefly, after de-waxing, rehydration, and blocking, heat-induced antigen retrieval was performed using Antigen Unmasking Solution (#H3300 [Vector Laboratories, Burlingame, CA], 1:100 in distilled water) in a Tefal pressure cooker (Tefal, Rumilly, France) for 10 minutes. The staining of the sections was performed using Biogenex Super-sensitive polymer – HRP kit (#QD440-XAKE; Biogenex, San Ramon, CA). Tissue sections were incubated with rabbit or mouse primary antibody (Table 3), optimally diluted in Zytomed Antibody diluent (#ZUC025-100; Zytomed, Berlin, Germany) for 40 minutes, washed, incubated with Super Enhancer Reagent for 20 minutes, and then with SS-label for 30 minutes. Vector VIP or DAB (3,3-diaminobenzidine) reagents were applied on the sections for 10 minutes, counterstained in hematoxylin (Gill’s II), dehydrated and mounted with DPX xylene-based permanent mountant, and left to dry. A scan of the entire section was taken using Pannoramic SCAN (3DHISTECH, Budapest, Hungary). After the slides had been scanned for the first staining, the coverslip was removed in xylene overnight. Antibodies were stripped from the sections using a second antigen retrieval step before staining with a second primary antibody. Immunohistochemistry-stained images were converted in pseudo-color images and overlaid using ImageJ (v1.53k National Institutes of Health, Bethesda, MD).

**Table 3:** Primary Antibodies for Immunohistochemistry

Species Raised in	Antibody	Company	Catalog Number
Mouse	Bcl-6	Dako	M7211
Mouse	CD20	Dako	M0755
Mouse	CD4	Novocastra	NCL-CD4-368
Mouse	CD68 (PGM1)	Dako	M0876
Mouse	CD8	Dako	M7103
Rabbit	DC-LAMP	Protein Atlas	HPA051467
Mouse	FDC (clone CNA.42) clone	Dako	M7157
Mouse	FOXP3	Abcam	20034
Mouse	Granzyme B	Dako	M7235
Mouse	TIA-1	Abcam	2712

### In Situ Hybridization

CXCL13 in situ hybridization was performed using RNAscope (#311329; Advanced Cell Diagnostics, Newark, CA) following the manufacturer’s instructions. A low-copy housekeeping gene probe (Polr2A, a DNA-directed RNA polymerase II subunit RPB1, #310451) and DapB gene (#310043) were used as positive and negative control probes, respectively.

### Criteria for TLS Definition in Human PDAC

Criteria for TLS identification are being debated.^47^ Our stringent criteria included presence of at least 3 cell types (CD3^+^ T cells, CD20^+^ B cells, and CD21^+^ FDCs) in a compact segregated structure with density of at least 0.1 TLS/mm^2^ (based on TLS abundance: high vs low or none). In addition, we demonstrate a critical mass of B cells (70 B cells/mm^2^) to discriminate between TLS^+^ and TLS^–^ patients.

### Animal Experiments

Male and female KPC mice, as described previously,^48^ were generated in house by crossing LSL (Lox-STOP-Lox) Kras^G12D/+^ and LSL-*Trp53*^*R172H/+*^ (C57BL/6/129/SVJae) with *Pdx-1-Cre* (C57BL/6) mice. The KPC-derived PDAC cell line (TB32048) generated from a female C57BL/6 KPC mouse (gift from the Tuveson Laboratory) was cultured for 3–4 passages at maximum 80% confluency in 10% fetal calf-serum (#A15-104; GE Healthcare, Milwaukee, WI) in Dulbecco’s modified Eagle medium (#E15-810; PAA) + 100μg/mL penicillin/streptomycin (#P11-G10; PAA) and tested negative for mycoplasma (#rep-pt1 [Invitrogen, Waltham, MA] and #LT07-710 [Lonza, Basel, Switzerland]). Cells were suspended in PBS and BD Matrigel Basement Membrane Matrix High Concentration (#354248; BD Biosciences, Franklin Lakes, NJ) at a ratio of 1:1. A total of 1000 cells in 10 μL were injected into the pancreas of 10- to 12-week-old female C57BL/6 mice (Charles River Laboratories, Edinburgh, United Kingdom) using a Hamilton syringe and size 22S gauge bevel tip needle (#10100332; Thermo Fisher Scientific, Waltham, MA).

Harvested tumors were cut along the sagittal and transverse planes in 4 halves: upper right and bottom left parts were fixed in formalin and embedded in paraffin or were placed in OCT, for immunostaining; upper left and bottom right were homogenized and used for flow cytometry analysis. Prior to tumor processing, lymph nodes and spleen were carefully excluded.

### Intratumoral Injection of Chemokines

Fresh aliquots of CXCL13 (#583906; BioLegend, San Diego, CA) and CCL21 (#586406; BioLegend) were used for each injection. A total of 2.5 μg of each chemokine, individually or combined, were injected in 20 μL PBS into orthotopic tumors, using an insulin syringe and a guided ultrasound Vevo2100 scanner (Visual Sonics, Toronto, Ontario, Canada) at predetermined time points for 3 cohorts according to availability of technicians: (1) days 20 or 21 and 25 (n = 4 mice each were treated with PBS, CXCL13, CCL21, or CXCL13/CCL21), (2) days 20 and 23 (n = 4 each for PBS and CXCL13/CCL21), and (3) days 20 and 24 (n = 8 each PBS and CXCL/13CCL21). Mice were culled at day 28. Mice that failed to grow pancreatic tumors were eliminated from the study (n = 4). Furthermore, from the main analysis (Figure 7) mice that developed a focal reaction (lymphoid aggregates) to injection-induced trauma were excluded from PBS- and chemokine-treated groups (n = 15). The phenotypic criteria for TLS identification, the B cell critical mass, and the mean value of %CD19^+^/CD45^+^ of the PBS-injected mice with lymphoid aggregate were used as a cutoff for discrimination between stress-induced lymphoid aggregate and potentially chemokine (CXCL13/CCL21)-induced TLSs.

Similarly, experiments were conducted for mice with or without gemcitabine (75 mg/kg) administered intraperitoneally as described previously,^49^ in which placebo control included intraperitoneal (for gemcitabine) or intratumoral (for chemokine) injection of PBS. For these experiments, 10 mice each were treated with PBS, CXCL13/CCL21 (#250-24, #250-13; PeproTech, Rocky Hill, NJ), gemcitabine, or combined (gemcitabine/CXCL13/CCL21) treatment. Injections (intratumoral chemokines and intraperitoneal gemcitabine) were performed from day 19, twice a week. In a first set of experiments, mice were culled at day 32. For the survival experiment, mice were treated twice a week for the lifespan of the animal, and the survival time (in days) was recorded. Pancreatic cancers were scanned and quantified by 3-dimensional ultrasonography twice a week and experiments were terminated if the primary tumor reached a maximum allowable dimension (length) of 1.85 cm or if a mouse showed signs of ill health. Tumor volume measurements were derived by the formula 4/3π(L/2∗D/2∗W2), where L is tumor length, D is tumor depth, and W is tumor width.

### Processing of Organs for Flow Cytometry

Murine tumors, after removal of adherent lymph nodes, and spleens were digested under agitation for 30 minutes in Dulbecco’s modified Eagle medium containing collagenase (2.0 mg/mL, #C9263; Sigma-Aldrich), DNAse (0.025 mg/mL, #D4513; Sigma-Aldrich) at 37°C, and passed through a 70-μm cell strainer (#11597522; Thermo Fisher Scientific) to achieve a single cell suspension. Human tumors were digested using the same media for up to 1 hour. Red blood cell lysis was performed for spleen and tumor samples using RBC Lyse buffer (#555899; BD Biosciences) for 10 minutes at RT. At 4°C, 0.5–2 × 10^6^ cells were incubated with anti-CD16/32 Fc Block (1:200, #553142; BD Biosciences) or Human TruStain FcX (#422301; BioLegend) for 15 minutes, followed by 50-μL Master Mix containing labeled antibodies (Table 4) for 30 minutes. Cells were then washed (fluorescence-activated cell sorting buffer: 5% bovine serum albumin, 2 mM EDTA, PBS), incubated with viability dye (FVD506, #65-0866; eBioscience, San Diego, CA) 20 minutes, washed and fixed in 2% paraformaldehyde (#158127, Sigma-Aldrich, Gillingham, UK) for 20 minutes. Subsequently, cells were washed and resuspended in fluorescence-activated cell sorting buffer for acquisition using the BD LSR Fortessa.

**Table 4:** Antibodies for Flow Cytometry

Antibody	Fluorochrome	Clone	Company	Catalog Number
CD11b	Alexa Fluor 700	M1/70	eBioscience	56-0112
CD11c	Brilliant Violet 650	N418	BioLegend	117339
CD19	PerCP-Cy5.5	eBio1D3	eBioscience	45-0193
CD19	FITC	eBIO1D3	eBioscience	11-0193-82
CD3	APC	17A2	BioLegend	100236
CD4	PerCP-Cy5.5	RM4-5	BioLegend	100540
CD45	Brilliant Violet 605	30-F11	BioLegend	103140
CD45R/B220	PE	RA3-6B2	eBioscience	12-0452
CD45R/B220	PerCP-Cy5.5	RA3-6B2	eBioscience	45-0452
CD86	Brilliant Violet 605	B7-2	BioLegend	105037
CD8a	Brilliant Violet 421	53-6.7	BioLegend	100738
F4/80	PE/Cy7	BM8	BioLegend	123114
FOXP3	APC	FJK-16s	eBioscience	17-5773-82
GL7	FITC	GL7	BioLegend	144604
Ly6C	e-Fluor 450	HK1.4	eBioscience	48-5932
Ly6G	PerCP-Cy5.5	1A8	BioLegend	127616
CD45 (a-human)	PE	2D1	BioLegend	368509
CD3 (a-human)	FITC	HIT3a	BioLegend	300306
CD19 (a-human)	BV605	HIB19	BioLegend	302244

For fluorescence-activated cell sorting, cells were stained as previous. A total of 100 μL of FcR block and 100 μL 2× antibody Master Mix was used per 10 × 10^6^ cells. The viability dye DAPI (#D9542; Sigma-Aldrich) was added at 2 μg/mL immediately before sample acquisition on the BD FACS Aria II. Samples were collected in 1 mL sterile fetal bovine serum.

### BMDC Co-Culture With B Cells

BMDCs were harvested from the tibiae and femurs of C57BL/6 mice flushed with RPMI and RBS lysis was performed. Bone marrow cells were plated in a T75 flask in RPMI with 10% heat-inactivated fetal bovine serum, 100-U/mL penicillin, 100-mg/mL streptomycin, 50-ng/mL granulocyte-macrophage colony-stimulating factor (#576304; BioLegend), and 50-ng/mL interleukin-4 (#574304; BioLegend) at 37°C in a humidified 5% CO_2_ atmosphere. On day 3, half of the media was removed and replaced with 2× granulocyte-macrophage colony-stimulating and 2× interleukin-4. On day 5, BMDCs were harvested, pelleted, and resuspended in fresh media supplemented with cytokines. On day 7, BMDCs were checked for their purity via flow cytometry using CD11c. BMDCs were plated overnight either in the presence or absence of tumor supernatant derived from KPC cell line TB32048, mixed in 1:1 with complete RPMI media. B cells were isolated from the spleen of healthy mice, or the spleen and tumor of KPC mice. All tumor B cells and some splenic B cells were isolated using flow sorting, as described previously, as CD45^+^ CD19^+^ cells. The remaining splenic B cells were isolated using B220 microbeads following the manufacturer’s protocol (#130-049-501; Miltenyi Biotec, Auburn, CA). B cells were seeded on top of BMDCs in a ratio of 4:1 in RPMI supplemented with 12.5 mM HEPES and 50 μM β-mercaptoethanol for 48 hours. Cells were then harvested using cell dissociation buffer (#13151-014; Gibco) and stained for CD86, for flow cytometry analysis (details in Table 4).

### Immunofluorescence Staining of Mouse Sections

All immunofluorescence was carried out at RT. Prior to staining, frozen samples were processed, generating 4- to 7-μm sections, which were stored at –80°C. For staining, frozen sections were warmed to RT for 10–300 minutes, fixed with 4% paraformaldehyde, #BX1143CB0201; Adams) or 1:1 methanol (#M/4056/17; Thermo Fisher Scientific) to acetone (#A/0520/17 [Thermo Fisher Scientific] or #CHE1040 [Scientific Laboratory Supplies, Nottingham, United Kingdom]) for 20 minutes at –20°C. Paraffin-embedded sections were deparaffinized, rehydrated, washed (0.1% PBS-T), permeabilized in (0.1% Triton, 5 minutes) and subjected to antigen retrieval (citrate buffer, pH 6), washed, blocked (blocking buffer with 0.02% fish gelatin, 5% goat serum, or #X0909 [Dako]; 30–360 minutes, RT) and incubated with primary unconjugated antibodies (Table 1) in blocking buffer or antibody diluent [#936B-08; Sigma-Aldrich], overnight at 4°C. Slides were then washed and incubated with secondary antibodies (Table 2). Conjugated primary antibodies, if needed, were added last for 1 hour. When required, slides were incubated with Sudan black for 3 minutes and washed 5× in PBS-T. Sections were counter-stained with DAPI (1:10,000 for 5 minutes) before being washed, mounted with Prolong Gold Antifade with DAPI (#P36931; Invitrogen) and stored at 4°C. Images were taken on the confocal microscope 510 and 710 (Zeiss) and processed using ImageJ (v1.53k). For TLS quantification, sections were scanned on the Axio Scan.Z1 (Zeiss) and NanoZoomer S60 (Hammamatsu Photonics) and analyzed using Zen, NDPview 2, ImageJ software, or QuPath.^50^

### Criteria for TLS Definition in Murine Models of PDAC

Similar to TLS criteria in human PDAC, murine TLSs were defined by immunostaining of sections for copresence of CD3^+^, B220^+^, FDC-M1^+^, or CD21^+^ cells in a compact organization. KPC and orthotopic implant tumors deemed negative for TLSs in initial assessment were reassessed 5 sections apart.

### Data Presentation and Statistical Analysis

Bright field images were analyzed using Pannoramic Viewer (3DHISTECH) software and Zen 2 (Zeiss). Immunofluorescence images were analyzed using Image J (Java) and NDP.view 2. Positively stained cells were automatically counted using the open source software QuPath.^50^ Intensity thresholds and parameters for cell detection and classification were set manually for each staining type and were identical for all samples.

Graphic representation of data and statistical analysis was performed using GraphPad Prism Version 8 (GraphPad Software, San Diego, CA). Data were tested for normality using the Kolmogorov-Smirnov test. If the data were normally distributed, then an unpaired *t* test or 1-way analysis of variance was used with Bonferroni's posttest. Nonparametric data were tested using a Mann-Whitney test or Kruskal-Wallis and Dunn's posttest or 2-sample Kolmogorov-Smirnov test. A chi-square test was used for TLS distribution data (positive/negative). Correlations were calculated using the Spearman rho test. Significance was established at *P <* .05.
